# Double Trouble: Flu Intensifies Effects of Ozone

**Published:** 2009-02

**Authors:** Cynthia Washam

Environmental health scientists have long speculated that the influenza virus could intensify the pulmonary effects of air pollution or vice versa. Like air pollution, influenza affects primarily the respiratory system, and ambient air pollutants may either lower resistance to viral infection or provide a vehicle that facilitates the spread of the virus, or both. There have been a number of laboratory-based animal studies on this potential relationship but no epidemiologic research. Researchers at the University of Hong Kong, in the first study of the influenza–air pollution interaction in humans, now report that respiratory hospitalizations and mortality significantly increased when ozone (O_3_) levels rose during flu season **[*EHP*** **117:248–253; Wong et al.]**.

The authors conducted a retrospective population-based study focusing on hospitalization and mortality rates for respiratory and cardiovascular disease. Medical data on patients diagnosed with respiratory or cardiovascular disease between 1996 and 2002 came from 14 Hong Kong hospitals. The authors determined “influenza intensity” during the same period as the percentage of respiratory specimens that tested positive for influenza each week. The Hong Kong Environmental Protection Department provided data on average daily concentrations of nitrogen dioxide (NO_2_), sulfur dioxide (SO_2_), particulate matter smaller than 10 μm (PM_10_), and O_3_.

As O_3_ levels increased during times of high influenza intensity, so did the number of hospitalizations and deaths from respiratory disease. The association was stronger in women than men, the researchers reported. There was no significant relationship between O_3_ and cardiovascular disease hospitalizations or mortality, and the data reflected no significant modification by influenza on the health effects of the other pollutants studied. Hong Kong has two flu seasons, peaking in January–February and May–July. O_3_ levels in Hong Kong typically peak in the sunniest months of September–December, when ultraviolet radiation reacts with nitrogen oxides and volatile organic compounds to form the noxious gas.

A surprising finding was a decrease in hospitalization for respiratory illness when peak PM_10_ concentrations coincided with flu outbreaks, whereas PM_10_ increases at other times were associated with increased hospitalizations. The researchers hypothesize that PM_10_ may diminish the flu effect by limiting the amount of ultraviolet light entering the atmosphere, which in turn would reduce the production of ozone.

The authors found weak interactions between influenza and both NO_2_ and SO_2_, but cautioned against drawing conclusions about individual pollutants that react in the atmosphere. NO_2_, for example, can combine with oxygen to form O_3_. The researchers propose that future studies focus on influenza’s potential interactions with a combination of pollutants in the atmosphere.

## Figures and Tables

**Figure:**
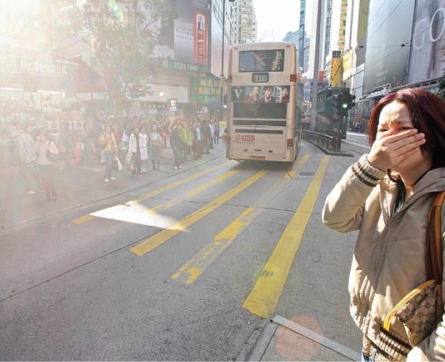
Influenza appeared to exacerbate the health effects of ozone pollution in Hong Kong.

